# Positive buttress reduction in femoral neck fractures: a literature review

**DOI:** 10.1186/s13018-024-04649-4

**Published:** 2024-04-24

**Authors:** Shun Han, Ze-Yang Zhang, Ke Zhou, Gui-Kun Yin, Yu-Chen Liu, Ben-jie Wang, Zhun Wen

**Affiliations:** 1 Department of Orthopaedic, Affiliated Zhongshan Hospital of Dalian University, Liaoning Province, Dalian 116001 P. R. China; 2 Department of Orthopaedic, Affiliated Xinhua Hospital of Dalian University, Liaoning Province, Dalian 116001 P. R. China; 3 Department of Orthopaedic, Central Hospital of Zhuanghe City, Zhuanghe, 116400 P. R. China

**Keywords:** Femoral neck fractures, Positive buttress reduction, Fracture reduction

## Abstract

**Background:**

Femoral neck fractures (FNFs) in young adults are usually caused by high-energy trauma, and their treatment remains a challenging issue for orthopedic surgeons. The quality of reduction is considered an important factor in improving the poor prognosis of patients with FNFs. In recent years, positive buttress closed reduction technique has received widespread attention in the treatment of FNFs. This comprehensive literature review is designed to encapsulate the impacts of both non-anatomic and anatomic reduction on the biomechanical stability, clinical outcomes, and postoperative complications in the management of FNFs, conjecture the efficacy of positively braced reduction techniques and provide a thorough summarization of the clinical outcomes.

**Methods:**

In this literature review, we have examined all clinical and biomechanical studies related to the treatment of FNFs using non-anatomical reduction or positive and negative buttress reduction. PubMed, Web of Science, Google Scholar and Embase Library databases were searched systematically for studies published before September 1, 2023. Published literature on fracture reduction techniques for treating FNFs was reviewed. In addition, we evaluated the included literature using the MINORs tool.

**Results:**

Although the “arch bridge” structure formed by the positive buttress reduction technique improved the support to the cortical bone and provided a more stable biomechanical structure, no significant differences were noted in the clinical efficacy and incidence of postoperative complications between the positive buttress reduction and anatomical reduction.

**Conclusion:**

Positive buttress reduction is an effective treatment method for young patients with FNFs. When facing difficult-to-reduce FNF, positive buttress reduction should be considered first, followed by anatomical reduction. However, negative buttress reduction should be avoided.

**Supplementary Information:**

The online version contains supplementary material available at 10.1186/s13018-024-04649-4.

## Introduction

As a result of an aging population, the number of people with hip fractures continues to increase and is expected to reach 63 million by 2050 [1, 2]. Most (54%) of the fractures occur in the neck of the femur [3]. The incidence of femoral neck fractures (FNFs) in middle-aged and young people is also on the increase [4]. FNFs in young adults are usually caused by high-energy trauma, which may involve displaced fracture patterns, leading to instability at the fracture site [5]. The treatment of these injuries remains a challenging issue for orthopedic surgeons [6, 7]. The vascular supply to bone is often damaged in displaced FNFs. As a result, displaced FNFs are often accompanied by a high rate of complications, including nonunion and osteonecrosis of the femoral head (ONFH) [8, 9].

The treatment of displaced FNFs often necessitates surgical intervention and comprehensive rehabilitation to restore mobility and function. Anatomical reduction is a common surgical technique used to realign fractured or dislocated bones and can be executed through an open or closed surgical approach. The fracture reduction technique aims to maximize the contact between the surface of the fractured ends to promote bone healing. In addition, it should also avoid excessive repeat reductions and the twisting of the intra-articular capsule artery to reduce the risk of damaging the blood supply [10–12]. However, the efficacy of this technique heavily relies on the surgeon’s experience. Moreover, anatomical reduction can not always be achieved, particularly in complex commuted fractures. Failure to achieve anatomical reduction can increase the risk of adverse events after surgery [13–16]. Unfortunately, postoperative complications such as nonunion, internal fixation failure, ONFH, infection, and nerve paralysis are common after FNFs reduction surgery, particularly in young people [17]. The risk of postoperative complications can increase if poor bone alignment is not detected. Poor alignment is more difficult to detect during closed reduction surgery. Immediate postoperative computed tomography scanning and three-dimensional reconstruction could be used to assess the quality of the reduction and reduce the risk of post-surgical complications. However, not all hospitals have the facilities to perform postoperative scanning.

**Fig. 1:**
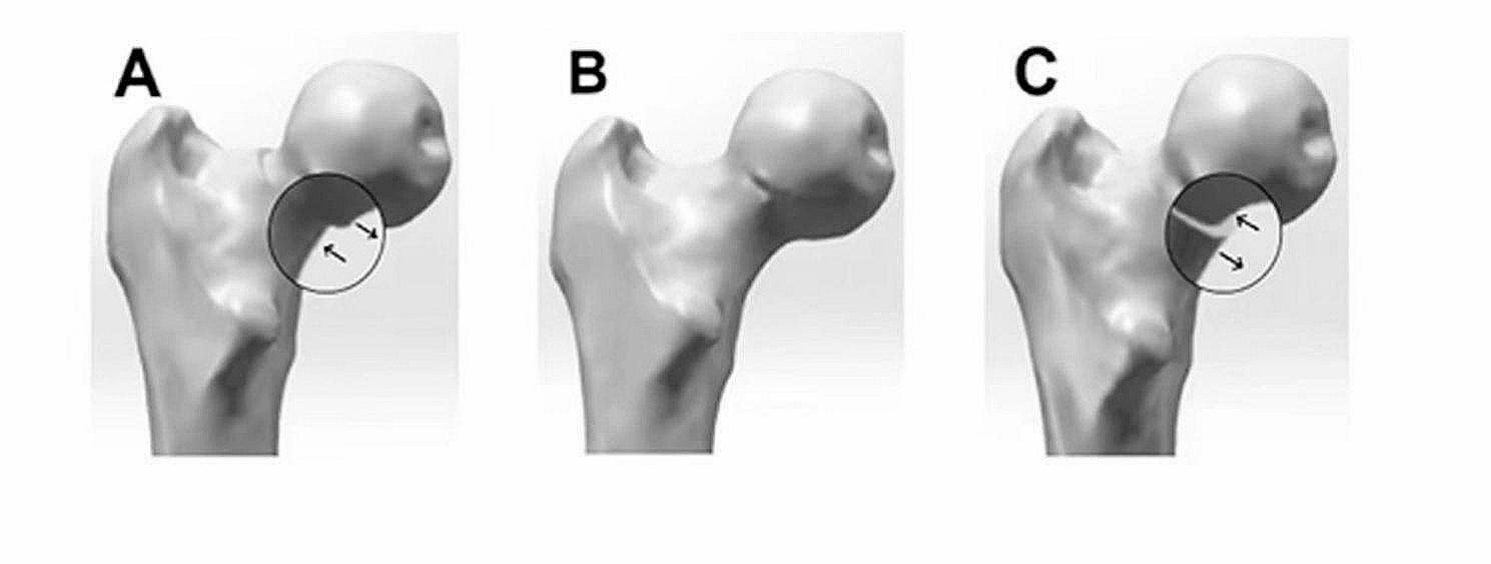
(**A**) negative buttress reduction, (**B**) anatomical reduction, and (**C**) positive buttress reduction

Non-anatomical reduction involves realigning the bone segments without necessarily restoring them to their original anatomical position. In 2012, Gotfried et al. introduced the non-anatomical closed positive and negative buttress reduction techniques to treat young patients with FNFs [18]. The term “positive buttress reduction mode” (Fig. 1C) refers to the situation where the proximal medial cortex of a FNF is located above the distal medial cortex on the outside, meaning that the distal medial cortex of the FNF protrudes towards the inner lower edge of the proximal end compared to an anatomical reduction (Fig. 1B). In contrast, in the “negative buttress reduction mode” (Fig. 1A), the proximal medial cortex of a FNF is located above the distal medial cortex on the inside, meaning that the proximal medial cortex of the FNF protrudes towards the inner upper edge of the distal end compared to an anatomical reduction (Fig. 1B). Intraoperative or postoperative anteroposterior X-rays of the hip joint are used to determine whether a positive reduction has been achieved [19]. However, to our knowledge, very few comprehensive literature reviews have been conducted evaluating the efficacy of the positive buttress reduction surgical technique for FNFs. Therefore, this comprehensive literature review is designed to encapsulate the impacts of both non-anatomic and anatomic reduction on the biomechanical stability, clinical outcomes, and postoperative complications in the management of FNFs, conjecture the efficacy of positively braced reduction techniques and provide a thorough summarization of the clinical outcomes.

## Materials and methods

### Literature search

**Fig. 2:**
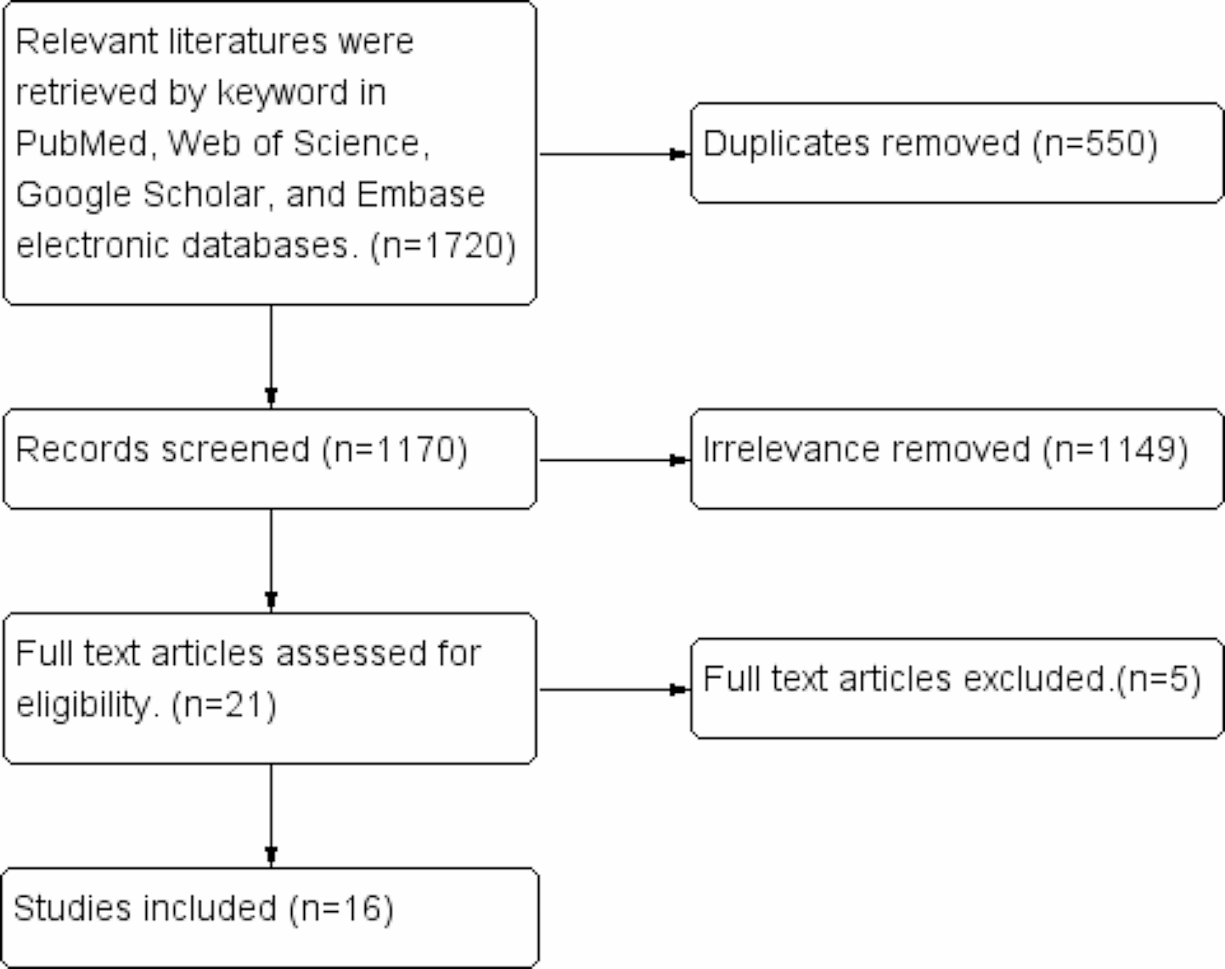
Literature screening flowchart

Details of the literature search process are shown in Fig. 2. The PubMed, Web of Science, Google Scholar, and Embase electronic database-s were searched to identify research articles comparing the biomechanical implants used to perform non-anatomical reduction for FNF and the clinical outcome of the different techniques. The following keywords were used to search for relevant articles “hip” OR “femur” OR “femoral” OR “femoral neck”) AND (fracture) AND (“anatomical” OR “anatomy” OR “positive” OR “negative” OR “Non-anatomical”) AND (reduction). All articles published before 2023 with no language restrictions were included in this literature review.

### Methodological quality of studies

The methodological quality of studies was assessed using the methodological index for non-randomized studies (MINORS) criteria [20], of which the first 8 criteria were used for all studies and all 12 for comparative studies. These outcomes are all displayed in Table 1. The level of evidence of all studies was assessed using the Oxford Centre for Evidence-Based Medicine (adjusted) [21].

**Table 1:** Summary of patient demographic data from the included studies

			Sample Size							
Study	Study Design	Implant	Total	AR	PBR	NBR	Age	Female Sex	Included Fracture Type	Follow-Up (months)
Ding et al.(2016)	RCS	CS	117	40	39	38	≤ 65	-		22.4
Lu et al.(2017)			105	35	35	35		30 (29%)	Garden I-IV	12
Tian et al.(2018)			96	48	48	0		34 (35%)		36
Xiong WF et al.(2019)			46	30	16	0		20 (43%)		22
Huang K et al.(2020)			67	21	24	22		28 (42%)		22.5
Zhao et al.(2021)			222	82	78	62		90 (40%)	Garden I-IV、Pauwels I-III	49.4
Zhao GL et al.(2021)			110	41	35	34		48 (44%)	Garden I-IV、Pauwels I-III	27
Yang et al.(2023)			74	28	25	21		38 (51%)	Garden I-IV	21.8
Zhu J et al.(2022)		DHS + DS	68	37	31	0		24 (35%)	Garden III/IV、Pauwels II/III	51.7
LI et al.(2022)	PCS	PCCP	69	23	23	23		22 (32%)	Garden I-IV	12
Jiang QL et al.(2023)	RCS	FNS	58	21	19	18		27 (47%)	Garden I-IV、Pauwels I-III	18.6
Total			1032	406	373	253				

AR, anatomical reduction; PBR, positive buttress reduction; NBR, negative buttress reduction; RCS, retrospective comparative study; PCS, prospective comparative research; CS, cannulated screw; DHS + DS, dynamic hip screw and derotation screw; PCCP, percutaneous compression plate; FNS, femoral neck system

## Results

### Characteristics of the studies

A total of 16 relevant studies were retrieved, of which 11 evaluated the clinical efficacy and 6 evaluated the biomechanics. Most studies evaluated the clinical efficacy of different reduction techniques in young patients (< 65 years old) with FNFs during and after closed reduction and internal fixation. Various implants were used to fix the fractures, including percutaneous compression plates (PCCP), cannulated screws (CS), dynamic hip screws (DHS) and derotation screws (DS), and femoral neck system (FNS). In the study of biomechanics, the implants model included cannulated screws (CS), dynamic hip screws (DHS) and derotation screws (DS), femoral neck system (FNS), and physiological hip nail (PHN) by three-dimensional finite element modeling. The summary information is presented in Tables 2 and 3.

**Table 2:** Data for harris hip score and complications rate from the included studies

Study	Harris hip score (follow up 1 year)			Femoral head necrosis			Shortening (> 5 mm) or Displacement to varus (> 10°)			Fracture nonunion		
	AR	PBR	NBR	AR	PBR	NBR	AR	PBR	NBR	AR	PBR	NBR
Ding et al.(2016)	84.5	86	78.9	15.0%	15.4%	18.4%	7.5%	7.7%	26.8%	NS		
Lu et al.(2017)	86.2	86.2	76.9	11.0%	11.0%	14.3%	14.2%	8.7%	25.7%	NS		
Xiong WF et al.(2019)	NS			3.3%	0	-	10.0%	6.4	-	6.7%	3.3%	-
Huang K et al.(2020)	85.6	84.5	74.3	19.1%	20.8%	22.7%	23.8%	41.7%	50.0%	NS		
Zhao et al.(2021)	85.9	85.4	81.9	13.4%	5.4%	32.2%	3.7%	5.1%	14.5%	3.7%	2.6%	12.9%
Zhao GL et al.(2021)	NS			12.2%	11.4%	20.6%	12.2%/4.9%	11.4%/5.7%	32.4%/11.8%	4.9%	2.9%	5.9%
Yang et al.(2023)	88.8	88.1	83.3	3.6%	4.0%	19.0%	21.4%	28%	66.7%	NS		
Jiang QL et al.(2023)	85.9	86.2	85.2	4.8%	5.3%	25.0%	9.5%	5.3%	16.7%	NS		
	Harris hip score ( good excellent rate)											
Tian et al.(2018)	89.6%	93.8%	-	16.7%	4.17%	-	NS			4.2%	0%	-
Zhu J et al.(2022)	64.8%	83.9%	-	10.8%	6.5%	-	PBR was lower than AR			5.4%	0%	-
LI et al.(2022)	100%	95.7%	64.3%	13.0%	8.7%	8.7%	13.04%	8.7%	39.1%	4.4%	8.7%	8.7%

AR, anatomical reduction; PBR, positive buttress reduction; NBR, negative buttress reduction

**Table 3:** Basic information and biomechanical experimental data of the included studies

Study	Implant	Fracture Type	Model Classification		The Peak displacement	The Peak Stress
2017 Zheng	PHN	Pauwels type III(70°)	4 Models (PBR and NBR under two loading conditions imulating “stance” and“walking” respectively)		The fracture end under “stance” conditions: 0.87 mm (PBR) and 1.38 mm (NBR), “walking” conditions:1.98 mm (PBR) and 1.27 mm(NBR)	The implants under“stance”conditions: 304.47 MPa (PBR) and 359.03 MPa (NBR),“walking”conditions:362.24 MPa (PBR) and 391.52 MPa (NBR)
2021 Zhao	CS	Pauwels type II(50°)	3 Models(AR、PBR、NBR)		The average displacement of fracture end: PBR < NBR	The mean stress of the calcar: PBR < NBR
2019 Wang	CS	Pauwels type I(30°)	5 Models (AR、PBR(2、3、4 mm) and NBR)		The fracture end: 0.547 mm(PBR 2 mm)、0.721 mm (PBR 3 mm) 、0.838 mm (PBR 4 mm) 、0.388 mm (AR) and 0.786 mm (NBR 2 mm)	The implants: 358.2Mpa (PBR 2 mm)、526.4Mpa (PBR 3 mm)、916.1Mpa (PBR 4 mm)、261.2Mpa (AR) and 705.8Mpa (NBR 2 mm)
2022 Zhu	DHS + DS	Pauwels type III(> 70°)	2 Models (AR、PBR)		The fracture end: PBR < AR	The implants: 360 MPa(PBR) < 515 MPa(AR)
2023 Fan Z	FNS	Pauwels type I(30°)	Total:18	9 Models: AR PBR(1、2、3、4 mm) NBR(1、2、3、4 mm)	1、The implants: 2.231 mm(AR) 2.229 mm(PBR 1 mm)、2.227 mm (PBR 2 mm) 、2.225 mm (PBR 3 mm) 、2.227 mm (PBR 4 mm) 、 2.233 mm(NBR 1 mm)、2.235 mm (NBR 2 mm) 、2.236 mm (NBR 3 mm) 、2.237 mm (NBR 4 mm) 。 2、The femur: 2.67 mm(AR) 2.466 mm(PBR 1 mm)、 2.466mma (PBR 2 mm) 、2.467 mm (PBR 3 mm) 、2.473 mm (PBR 4 mm) 、 2.467 mm(NBR 1 mm)、2.467 mm (NBR 2 mm) 、2.467 mm (NBR 3 mm) 、 2.466 mm (NBR 4 mm) 。	1、The implants: 432.4 MPa(AR) 430.7 MPa(PBR 1 mm)、429.7 MPa (PBR 2 mm) 、542.4 MPa (PBR 3 mm) 、536.3 MPa (PBR 4 mm) 、 801.6Mpa(NBR 1 mm)、800.3Mpa (NBR 2 mm) 、540.5Mpa (NBR 3 mm) 、539.1Mpa (NBR 4 mm) 。 2、The femur: 85.97 MPa(AR) 89.51 MPa(PBR 1 mm)、94.57 MPa (PBR 2 mm) 、88.75 MPa (PBR 3 mm) 、76.44 MPa (PBR 4 mm) 、 89.11Mpa(NBR 1 mm)、89.45Mpa (NBR 2 mm) 、 89.38Mpa(NBR 3 mm)、88.56Mpa (NBR 4 mm) 。
2023 Fan Z	FNS	Pauwels type II(50°)	Total:18	9 Models: AR PBR(1、2、3、4 mm) NBR(1、2、3、4 mm)	1、The implants: 2.288 mm(AR) 2.302 mm(PBR 1 mm)、2.340 mm (PBR 2 mm) 、 2.390 mm(PBR 3 mm)、2.415 mm (PBR 4 mm) 、 2.286 mm(NBR 1 mm)、2.293 mm (NBR 2 mm) 、 2.320 mm(NBR 3 mm)、2.335 mm (NBR 4 mm) 。 2、The femur: 2.533 mm(AR) 2.562 mm(PBR 1 mm)、2.621 mm (PBR 2 mm) 、 2.693 mm(PBR 3 mm)、2.736 mm (PBR 4 mm) 、 2.520 mm(NBR 1 mm)、2.518 mm (NBR 2 mm) 、 2.543 mm(NBR 3 mm)、2.552 mm (NBR 4 mm) 。	1、The implants: 514.6 MPa(AR) 685 MPa(PBR 1 mm)、757.7 MPa (PBR 2 mm) 、 843.5 MPa(PBR 3 mm)、880.4 MPa (PBR 4 mm) 、 660.4Mpa(NBR 1 mm)、678.1Mpa (NBR 2 mm) 、 730.9Mpa(NBR 3 mm)、759.2Mpa (NBR 4 mm) 。 2、The femur: 95.63 MPa(AR) 114.6 MPa(PBR 1 mm)、126.1 MPa (PBR 2 mm) 、 99.94 MPa(PBR 3 mm)、88.89 MPa (PBR 4 mm) 、 86.83Mpa(NBR 1 mm)、247.7Mpa (NBR 2 mm) 、 184.6Mpa(NBR 3 mm)、182.5Mpa (NBR 4 mm) 。
2023 Jia	FNS	Pauwels type I(30°)	Total:9	3 Models (AR、PBR(2 mm)、NBR(2 mm))	1、The implants: 1.1324 mm(PBR 2 mm)、 1.1712 mm(AR)and 1.220 mm (NBR 2 mm) 2、The femur: 1.2881 mm(PBR 2 mm)、 1.3387 mm(AR)and 1.4052 mm (NBR 2 mm)	1、The implants: 286.66Mpa(PBR 2 mm)、 323.98Mpa(AR)and 374.58Mpa (NBR 2 mm) 2、The femur: 64.07Mpa(PBR 2 mm)、 65.485Mpa(AR)and 79.271Mpa (NBR 2 mm)
		Pauwels type II(50°)		3 Models (AR、PBR(2 mm)、NBR(2 mm))	1、The implants: 1.1485 mm(PBR 2 mm)、1.1712 mm (AR) and 1.1746 mm (NBR 2 mm) 2、The femur: 1.312 mm(PBR 2 mm)、1.3355 mm (AR) and 1.4068 mm (NBR 2 mm)	1、The implants: 303.56Mpa(PBR 2 mm)、328.05Mpa (AR) and 429.45Mpa (NBR 2 mm) 2、The femur: 65.523Mpa(PBR 2 mm)、66.767Mpa (AR) and 79.516Mpa (NBR 3 mm)
		Pauwels type III(70°)		3 Models (AR、PBR(2 mm)、NBR(2 mm))	1、The implants: 1.2036 mm(PBR 2 mm)、1.2089 mm (AR) and 1.245 mm (NBR 2 mm) 2、The femur: 1.3919 mm(PBR 2 mm)、1.3777 mm (AR) and 1.4068 mm (NBR 2 mm)	1、The implants: 330.19Mpa(PBR 2 mm)、331.15Mpa (AR) and 383.18Mpa (NBR 2 mm) 2、The femur: 112.19Mpa(PBR 2 mm)、105.94Mpa (AR) and 142.43Mpa (NBR 4 mm)

AR, anatomical reduction; PBR, positive buttress reduction; NBR, negative buttress reduction; CS, cannulated screw; DHS + DS, dynamic hip screw and derotation screw; FNS, femoral neck system;

PHN, Physiological Hip Nail

### Metrics used to assess the clinical outcomes post-surgery

The incidences of complications post-surgery, including ONFH, shortening (femoral neck shortening exceeding 5 cm [22]) and displacement (changes in neck-shaft angle exceeding 10° [23]) of the femoral neck, nonunion, infection, and postoperative fractures, were assessed in most studies. In addition, most studies used the Harris Hip score to evaluate the outcomes and function of the patient’s hip joint after surgery [24]. The Harris Hip Score consists of a series of questions and physical assessments, with a total score ranging from 0 to 100 points. Higher scores indicate better hip function and less pain.

### Hip function score post-surgery

A total of 11 studies, including 1032 young patients with unilateral FNF, were evaluated. Among them, 373 had positive buttress reduction, 406 had anatomical reduction, and 253 had negative buttress reduction. Table 4 provide a summary of the postoperative complications. The majority of the patients were followed up for more than one year. None of the studies identified a statistical difference in the Harris hip score one year after surgery between patients treated with positive buttress reduction and anatomical reduction [25]. However, in some studies, patients treated with positive buttress reduction had a higher rate of excellent Harris scores (> 80 points) than those treated with anatomical reduction [26, 27] (*P* < 0.05). The patients treated with positive buttress reduction and anatomical reduction had a better Harris hip score than those treated with negative buttress reduction (*P* < 0.05) [28–33].

**Table 4:** Quality assessment of the included studies using the MINORS criteria^a^

First Author	Year	Journal	LoE	Study design	MINORS Criteria^b^												
					1	2	3	4	5	6	7	8	9	10	11	12	Total
Ding et al.	2016	*Chin J Orthop Trauma*	3	RCS	2	2	0	2	0	2	2	0	2	2	2	1	17
Lu et al.	2017	*China J. Mod. Med.*	3		2	2	0	2	0	2	2	0	2	2	2	1	17
Tian et al.	2018	*China J. Mod. Med.*	3		2	2	0	2	0	2	2	0	2	2	2	1	17
Xiong WF et al.	2019	*J ORTHOP SURG RES*	3		2	2	0	2	0	2	2	0	2	2	2	1	17
Huang K et al.	2020	*J ORTHOP SURG RES*	3		2	2	0	2	0	2	2	0	2	2	2	1	17
Zhao et al.	2021	*J ORTHOP SURG RES*	3		2	2	0	2	0	2	2	0	2	2	2	1	17
Zhao GL et al.	2021	*BioMed Res. Int.*	3		2	2	0	2	0	2	2	0	2	2	2	2	18
Yang et al.	2023	*Chin. J. Repar. Reconstr. Surg.*	3		2	2	0	2	0	2	2	0	2	2	2	2	18
Zhu J et al.	2022	*BioMed Res. Int.*	3		2	2	0	2	0	2	2	0	2	2	2	2	18
LI et al.	2022	*JMMC*	3	PCS	2	2	2	2	1	2	2	1	2	2	2	1	21
Jiang QL et al.	2023	*BMC Musculoskelet Disord*	3	RCS	2	2	0	2	0	2	2	0	2	2	2	2	18

^a^LoE, level of evidence; MINORS, methodological index for non-randomized studies. Blank cells indicate not applicable

^b^MINORS criteria [20]: 0 points when not reported, 1 when reported but not adequate, and 2 when reported and adequate; maximum score, 24 for comparative studies [1]. A clearly stated aim: the question addressed should be precise and relevant in the light of available literature [2]. Inclusion of consecutive patients: all patients potentially fit for inclusion (satisfying the criteria for inclusion) have been included in the study during the study period (no exclusion or details about the reasons for exclusion) [3]. Prospective collection of data: data were collected according to a protocol established before the beginning of the study [4]. Endpoints appropriate to the aim of the study: unambiguous explanation of the criteria used to evaluate the main outcome, which should be in accordance with the question addressed by the study. In addition, the endpoints should be assessed on an intention-to-treat basis [5]. Unbiased assessment of the study endpoint: blind evaluation of objective endpoints and double-blind evaluation of subjective endpoints. Otherwise, the reasons for not blinding should be stated [6]. Follow-up period appropriate to the aim of the study: the follow-up should be sufficiently long to allow the assessment of the main endpoint and possible adverse events [7]. Loss to follow-up\5%: all patients should be included in the follow-up. Otherwise, the proportion lost to follow-up should not exceed the proportion experiencing the major endpoint [8]. Prospective calculation of the study size: information of the size of detectable difference of interest with a calculation of95%CI, according to the expected incidence of the outcome event, and information about the level for statistical significance and estimates of power when comparing the outcomes [9]. An adequate control group: having a gold standard diagnostic test or therapeutic intervention recognized as the optimal intervention according to the available published data [10]. Contemporary groups: control and studies group should be managed during the same period [11]. Baseline equivalence of groups: the groups should be similar regarding the criteria other than the studied endpoint. Absence of confounding factors that could bias the interpretation of the results [12]. Adequate statistical analyses: whether statistics were in accordance with type of study with calculation of confidence intervals or relative risk

### Incidence of postoperative complications

Most research results found no significant difference in the incidence of complications between the positive buttress reduction group and the anatomical reduction group (*P* > 0.05). Conversely, the negative buttress reduction group had a significantly higher incidence of postoperative complications than the positive buttress reduction and anatomical reduction groups (*P* > 0.05) [23, 28–35]. Some research studies reported a lower incidence of ONFH, shortening and displacement of the femoral neck, and fracture nonunion complications in the positive buttress reduction group when compared with the anatomical reduction group [26, 27]. However, it’s important to note that the difference in the incidence of fracture nonunion was not statistically significant between the 2 groups, possibly due to the limited sample size. (*P* > 0.05) [23, 26, 27, 31, 34, 35]. The summary information is presented in Table 4.

### Biomechanical evaluation

The postoperative effect is inseparable from the biomechanical performance of the internal fixator. At present, most biomechanical studies use finite element analysis, which directly reflects the stability of the model by measuring the maximum displacement value and maximum stress value of the fracture end under external load. The smaller the displacement value, the more solid the fixation [36]. The stress cloud map can reflect the situation of stress transmission when force is applied to the corresponding part. The summary information is presented in Table 3.

Although the internal fixation methods used to develop biomechanical 3D models varied widely between studies, they all reached similar conclusions. Compared to negative buttress reduction, the positive buttress reduction technique resulted in better stability, stress transmission, biomechanical performance, and safer internal fixation [37, 38]. However, there is still controversy about whether positive buttress reduction or anatomical reduction is better. So far, biomechanical performance studies comparing positive buttress reduction in relation to anatomical reduction showed that the biomechanical performance brought by positive buttress reduction (displacement 0-2 mm) is closest to anatomical reduction [38–40]. In the positive buttress reduction (displacement 2 mm ) mode, the screws bear less stress, indicating that the medial cortex can disperse some screw stress in positive buttress reduction mode [39, 41]. If the displacement is too large, it will weaken the mechanical advantage of positive buttress mode and even approach negative buttress [38–40].

Wang et al. [39] proposed a four-tier classification to guide positive buttress reduction mode based on the extent of displacement whereby grade 1 includes displacement from 0 to 2 mm, grade II includes displacement in the range of 2–3 mm, grade III includes displacement ranging from 3 to 4 mm and grade IV includes displacement exceeding 4 mm. Studies have shown that in cases where anatomical reduction is not feasible, positive buttress reduction grade I can achieve biomechanical effects similar to anatomical reduction for FNF. And then positive buttress reduction grade II is a relatively acceptable range. However, the use of positive buttress reduction Grade III and IV for displaced FNF is not recommended. In addition, Wang et al. [42] found that Gotfried positive buttress reduction was more effective than open precision reduction and Gotfried negative buttress reduction for bone healing and blood supply recovery in rabbits with FNFs, but the bone growth capacity of open precision reduction is greater than that of Gotfried positive buttress reduction.

Jia et al. [38] and Fan et al. [40] showed that the biomechanical performance of positive buttress reduction was also related to the angle of inclination of the FNF in relation to the femoral shaft, also known as the Pauwels angle. For Pauwels type I fractures (below 30°), the biomechanical performance of positive buttress reduction was very close to that of the anatomical reduction. However, as the Pauwels angle increases, the mechanical performance of positive buttress reduction gradually weakens [38, 40]. Eventually, for Pauwels type III fractures (above 70°), anatomical reduction provided better stability than positive buttress reduction.

## Discussion

Anatomical reduction and rigid internal fixation have been considered the treatment of choice for decades for young patients (below 65 years) with displaced and unstable FNFs [43, 44]. However, in cases of complex commuted fractures, closed surgical anatomical reduction is not always possible [39]. Positive buttress reduction can provide an alternative reasoning to the reduction of FNF. However, it is important to note that despite the growing interest in the Gotfried positive buttress reduction technique, there appears to be a noticeable gap in comprehensive literature reviews and systematic evaluations of its clinical efficacy and biomechanical stability. Therefore, in this literature review, we aimed to evaluate the clinical efficacy and biomechanical properties of the positive buttress reduction technique in relation to anatomical reduction techniques for FNF.

### Development and clinical efficacy of the positive buttress reduction method

The objective of Gotfried positive buttress reduction is to align the bones to attain a line measuring between 160° to 180° on the hip joint lateral X-ray, with both the proximal and distal fracture ends aligning with the positive buttress position on the hip joint anterior X-ray. Simultaneously, the femoral neck-shaft angle should demonstrate a minimum of 135° with external rotation. Studies have found [38–40] that the biomechanical performance, safety of the internal fixator implantation, and reliability of the postoperative fracture alignment of positive buttress reduction (displacements ranging between 0 and 2 mm) are similar to those obtained following anatomical reduction. Positive buttress reduction with a displacement within 2 mm joint a fixed nail system can provide stable mechanical fixation in displaced FNF that can not be fixed with anatomical reduction. However, negative buttress reduction should be avoided whenever possible. Moreover, compared with anatomical reduction, the positive buttress reduction technique has demonstrated favorable clinical outcomes, characterized by swift recovery of hip joint function and a reduced or comparable incidence of postoperative complications, including femoral neck shortening and ONFH. Consequently, based on the findings of this literature review we suggest that, for FNF, positive buttress reduction can be the first choice, followed by anatomical reduction. Conversely, using negative buttress reduction is discouraged, and patients should receive dependable internal fixation instead.

### Stability of the positive buttress reduction post-surgery

Irrespective of the quality of the anatomical reduction, during the healing process, bone absorption and shear force at the fracture site may still cause secondary sliding and displacement, leading to shortening of the femoral neck and reduction of the neck-shaft angle. It is well known that an important predictive indicator of failure after surgery is the bending displacement of the femoral neck [45]. Positive buttress reduction can effectively avoid the negative effects of bone absorption and shear force by improving the bone support at the fracture site. During the positive buttress reduction procedure, the inner cortex of the proximal head and neck bone block of the fracture is positioned on the outer and upper side of the inner cortex of the distal fracture. A lateral displacement is then applied so that the cortices at both ends of the fracture come into contact with each other to eventually form a small arch-like step that helps distribute some of the stress from above. Additionally, the head and neck region receives added support from the inner cortex of the femoral neck, thus reducing excessive displacement of the proximal fracture end. These arrangements eventually maintain a stable cortical-to-cortical configuration, reducing the risk of bone displacement post-surgery [41]. Conversely, during anatomical reduction, the head and neck fragments are only supported by fixation screws, and no support is received from the inner cortex of the femoral neck. As a result, positive buttress reduction can establish a more stable structural alignment and reduce the risk of femoral neck shortening while preserving the neck-shaft angle.

### Adaptation of the positive buttress reduction technique based on fracture location

In FNFs or intertrochanteric fractures, positive buttress reduction has a different application. A prerequisite for the performance of Gotfried positive buttress reduction is a head and neck bone block located on the outside of the inner cortex of the distal femur. However, while this approach works well for FNF, it may not be suitable for intertrochanteric fractures. In view of this, Zhang et al. [46] first proposed that in the reduction of intertrochanteric fractures, the position of the buttresses is altered so that the inner cortex of the proximal head and neck bone block is situated on the inside of the inner cortex of the distal femur to form the positive buttress. Conversely, the inner cortex of the proximal head and neck bone block is positioned on the outside of the inner cortex of the distal femur to form the negative buttress. Moreover, it is important to note that the mechanical forces of the hip post-surgery vary between FNF and intertrochanteric fractures [47, 48]. In FNF, the vertical shear force is the main factor affecting fracture stability. In contrast, the shear force and hip joint internal rotation coexist in intertrochanteric fractures due to the long proximal lever arm. Therefore, in order to obtain a secondary stable sitting at the fracture end, the surgical management of these 2 types of fractures requires different strategies. In FNF, an uplifting force should be applied to the proximal cortical bone against the distal cortical bone to prevent downward movement. This technique is known as uplifting reduction. However, for intertrochanteric fractures, a push-out force should be applied to the proximal cortical bone to prevent inward displacement of the proximal bone block. This approach is known as push-out reduction.

## Conclusion

The Gotfried positive buttress reduction mode is an effective treatment strategy for young patients with FNF. However, most of the current clinical efficacy analysis studies on positive buttress reduction are based on small retrospective studies with a primary emphasis on using hollow nails as the chosen internal fixation method in positive buttress reduction procedures. Therefore, larger prospective multicenter studies are required to confirm the efficacy of this technique. Moreover, additional research is required to compare the efficacy of different fixation methods.

### Electronic supplementary material

Below is the link to the electronic supplementary material.

Supplementary Material 1

## Data Availability

Not applicable.

## Abbreviations

**PBR**: positive buttress reduction
**AR**: anatomical reduction
**NBR**: negative buttress reduction
**FNFs**: femoral neck fractures
**ONFH**: osteonecrosis of the femoral head
**DHS**: dynamic hip screws
**CS**: cannulated screws
**PCCP**: percutaneous compression plates
**DS**: derotation screws
**FNS**: femoral neck system
**PHN**: physiological hip nail
