# Supplementary material for: Positive buttress reduction in femoral neck fractures: a literature review
Source: J Orthop Surg Res. 2024 Apr 24;19:262. doi: 10.1186/s13018-024-04649-4 (PMC11044489; doi:10.1186/s13018-024-04649-4)
Supplement: Supplementary file 1 — Supplementary Material 1 [file 13018_2024_4649_MOESM1_ESM.docx]

**Positive Buttress Reduction in Femoral Neck Fractures: A** **Literature Review**

**Authors:** Shun Han^1, #^, Ze-Yang Zhang^1, #^, Ke Zhou^1^ ,Gui-Kun Yin^3^, Yu-Chen Liu^1^, Ben-jie Wang^2, *^, Zhun Wen^3, *^

^1.^Department of Orthopaedic, Affiliated Zhongshan Hospital of Dalian University, Dalian, 116001, Liaoning Province, P. R. China

^2^^.^Department of Orthopaedic, Affiliated Xinhua Hospital of Dalian University, Dalian, 116001, Liaoning Province, P. R. China

^3.^Department of Orthopaedic, Central Hospital of Zhuanghe City, Zhuanghe, 116400, Liaoning Province, P. R. China

*** Corresponding author:**

Dr Ben-jie Wang, Department of Orthopaedic, Affiliated Zhongshan Hospital of Dalian University, Dalian, Liaoning 116001, P.R. China, E-mail: wangbenjie@dlu.edu.cn

Dr Zhun Wen, Department of Orthopaedic, Central Hospital of Zhuanghe City, Zhuanghe, 116400, Liaoning Province, P. R. China, E-mail: yinguikun@163.com

^#^ These authors contributed equally to this work and should be considered co-first authors.

**Keywords:** femoral neck fractures; Positive buttress reduction; fracture reduction;

**Running Title:** The clinical efficacy and biomechanics of the PBR

**E-mail addresses:** hanshun0505@163.com(S.Han),zhangzeyang0502@163.com(Z.Zhang),no1_good@163.com(K.Zhou),h120455@outlook.com(G.Yin),liuyuchen2021042@163.com(Y.Liu),yinguikun@163.com(Z,Wen),wangbenjie@dlu.edu.cn(B,Wang)

**Authorship declaration.**

i) that all authors listed meet the authorship criteria according to the latest guidelines of the International Committee of Medical Journal Editors, and ii) that all authors are in agreement with the manuscript.

**Ethics approval and consent to participate**

This study was performed in line with the principles of the Declaration of Helsinki. Approval was granted by the Ethics Committee of Affiliated Zhongshan Hospital of Dalian University.

**Consent for publication**

Not applicable

**Availability of data and materials statement**

Not applicable

**Competing interests**

The authors declare that they have no competing interests

**Funding**

Not applicable

**Authors' contributions**

S-H performed a literature search and was a major contributor in writing the manuscript; ZY-Z was a major contributor in writing the manuscript; K-Z, GK-Y, and YC-L were contributors in writing the manuscript; BJ-W and Z-W was responsible for reviewing and editing the manuscript. All authors read and approved the final manuscript.

**Acknowledgements**

We are grateful to all participating patients.
